# Comments from the Editor of the Special Issue “Type 2 Diabetes: Update on Pathophysiology and Treatment”

**DOI:** 10.3390/jcm8111939

**Published:** 2019-11-11

**Authors:** Tomoaki Morioka

**Affiliations:** Department of Metabolism, Endocrinology, and Molecular Medicine, Osaka City University Graduate School of Medicine, Osaka 545-8585, Japan; m-tomo@med.osaka-cu.ac.jp; Tel.: +81-66-645-3806; Fax: +81-66-645-3808

**Keywords:** type 2 diabetes, obesity, kidney disease, cancer, elderly, microbiota, microRNA, deep neural network, glucagon-like peptide-1, dementia, bariatric surgery

## Abstract

The editor would like to offer the Special Issue “Type 2 Diabetes: Update on Pathophysiology and Treatment” to the community of diabetes clinicians and researchers. This Special Issue aims to update our understanding of the pathophysiology and treatment of type 2 diabetes and its vascular and non-vascular complications.

The prevalence of adult type 2 diabetes has greatly expanded in the last several decades, and about one in eleven adults worldwide are now estimated to have diabetes mellitus [1]. In addition, the prevalence of older adults with diabetes is growing especially in developed countries [2]. Recent data on trends in diabetes-related complications suggest a reduction in classic complications of type 2 diabetes and a diversification of diabetes morbidity, including long-term kidney disease, functional disability, sarcopenia, dementia, and cancer [3]. There is an urgent need to develop new treatment strategies against diabetes to combat this huge health problem. This Special Issue will expand our knowledge of the disease process of type 2 diabetes and its complications, and of the current status of comorbidities and treatment of diabetes worldwide, covering a wide variety of novelties that are being obtained from both basic and clinical research.

This Special Issue includes several articles providing new insights on the pathogenesis of type 2 diabetes. Flores-Guerrero et al. provide evidence from a prospective cohort study that high concentrations of plasma branched-chain amino acids are associated with insulin resistance and an increased risk of type 2 diabetes. Notably, the association is independent of established risk factors, insulin resistance, and β-cell function. Goto et al. identify a novel mechanism by which the mineralocorticoid receptor signaling exerts a protective role in pancreatic islets through the induction of interleukin-6 and glucagon-like peptide-1 (GLP-1) in α-cells.

In the field of genetics, Diaz-Morales et al. associate the mitochondrial DNA haplogroup (m.4216T>C) with poor glycemic control and impaired kidney function as compared to those with other haplogroups in Spanish type 2 diabetes patients. This finding suggests the mitochondrial DNA haplotype screening to be a way of predicting disease progression in newly diagnosed type 2 diabetes patients.

There are several interesting reports on epidemiology. Using the Korean health insurance database with over 3 million subjects, Park et al. find that bodyweight fluctuations over 4.4 years are an independent risk factor for new-onset diabetes after adjustment for possible confounders, including baseline BMI. This study underscores the importance of maintaining stable body weight for the prevention of diabetes. Hseish et al. utilize the health insurance database of Taiwan and develop a prediction model for colorectal cancer for patients with type 2 diabetes, using a deep neural network, which is superior to a single variable predictor using the adopted Diabetes Complication Severity Index.

A number of interesting reports on diabetes treatment are also included. Ghai et al. identify many type 2 diabetes-related miRNAs in the extracellular vesicle (EV), but not in whole or EV-depleted plasma, which decrease to levels close to healthy controls following metformin treatment. This finding suggests that miRNAs in EV may serve as biomarkers for the response to metformin intervention in patients with type 2 diabetes. Izaguirre et al. highlight the role of GLP-1 in limiting human adipocyte inflammation and a close association between pre-operative GLP-1 concentrations and remission of diabetes after Roux-en-Y gastric bypass. The report suggests a pre-operative GLP-1 concentration to be a predictor of diabetes remission after bariatric surgery in obese patients. Seghieri et al. compare β-cell function and glycemic control following a 6-week basal insulin treatment versus metformin in 38 newly diagnosed type 2 diabetes patients with mild obesity (mean BMI, 31 kg/m^2^) and mild hyperglycemia (mean HbA1c, 7.5%). The result indicates that a short course of basal insulin therapy does not offer any clinical advantage over recommended initiation with metformin in newly diagnosed patients. Milder et al. provide a meta-analysis of combination therapy with a sodium-glucose cotransporter 2 (SGLT2) inhibitor as an initial treatment for type 2 diabetes. The report indicates that initial SGLT2 inhibitor/metformin combination therapy, compared with either agent alone, has glycemic and weight benefits and appears safe and that a high-dose SGLT2 inhibitor/metformin combination has modest weight but not glycemic benefits compared with the low dose combination therapy.

In the field of diabetic complications, Miyake et al. highlight the importance of urinary albuminuria, regardless of the estimated glomerular filtration ratio, as a predictor of all-cause mortality in a historical cohort study of 385 Japanese patients with type 2 diabetes. Lin et al. analyze the metabolomic profiles of the cerebrospinal fluid by ^1^H nuclear magnetic resonance spectroscopy in type 2 diabetes versus controls and provide novel roles for metabolic dysregulation in cerebrospinal fluid as predictors of diabetic microangiopathy among type 2 diabetes patients.

Although not introduced here, many other articles in this Special Issue shed light on new findings in pathophysiology, genetics, epidemiology, treatment, and complications of type 2 diabetes. The editor hopes that these articles will help readers update their knowledge of type 2 diabetes research. Finally, the editor deeply appreciates all the authors for contributing excellent articles to this Special Issue. 
